# The Influence of Proximity to City Parks on Blood Pressure in Early Pregnancy 

**DOI:** 10.3390/ijerph110302958

**Published:** 2014-03-11

**Authors:** Regina Grazuleviciene, Audrius Dedele, Asta Danileviciute, Jone Vencloviene, Tomas Grazulevicius, Sandra Andrusaityte, Inga Uzdanaviciute, Mark J. Nieuwenhuijsen

**Affiliations:** 1 Department of Environmental Science, Vytautas Magnus University, K. Donelaicio G. 58, Kaunas, LT-44248, Lithuania; E-Mails: a.dedele@gmf.vdu.lt (A.D.); a.danileviciute@gmf.vdu.lt (A.D.); j.vencloviene@gmf.vdu.lt (J.V.); t.grazulevicius@gmail.com (T.G.); s.andrusaityte@gmf.vdu.lt (S.A.); i.uzdanaviciute@gmf.vdu.lt (I.U.); 2 Centre for Research in Environmental Epidemiology (CREAL), Doctor Aiguader 88, Barcelona 08003, Spain; E-Mail: mnieuwenhuijsen@creal.cat

**Keywords:** city park proximity, blood pressure groups, women, GIS, multinomial regression analysis

## Abstract

This study investigated the effect of proximity to city parks on blood pressure categories during the first trimester of pregnancy. This cross-sectional study included 3,416 female residents of the city of Kaunas, Lithuania, who were enrolled in the FP7 PHENOTYPE project study. The women were classified into four blood pressure categories: optimal, normal, high-normal blood pressure, and hypertension. Multinomial regression models were used to investigate the association between three women’s groups with respect to the residence distances from city parks (300, >300–1,000, and >1,000 m) and four blood pressure categories. When using the optimal blood pressure as the reference group, the crude and adjusted odds ratios (OR) for normal blood pressure and for high-normal blood pressure proved to be statistically significantly higher after the inclusion of the selected covariates into the regression analysis. The probability of normal blood pressure increased by 9%, and that of high-normal blood pressure—by 14% for every 300 m increase in the distance to green spaces. The findings of this study suggest a beneficial impact of nearby city parks on blood pressure amongst 20- to 45-year-old women. This relationship has important implications for the prevention of hypertension and the reduction of hypertension-related morbidity.

## 1. Introduction

Environmental exposures have long been suspected to have an independent influence on health outcomes, particularly in terms of cardiovascular disease and hypertension—the second leading cause of chronic diseases worldwide [1,2,3,4]. Blood pressure (BP) is affected by many environmental factors, including the living environment, social status, ambient temperature, altitude, noise, and air pollutants [5,6,7,8,9]. While most published studies have found that cardiovascular morbidity and hypertension may be related to the level of air pollution or neighbourhood quality [10,11,12,13,14,15,16], these findings are not consistent with respect to the role of environmental exposures and the magnitude of the effect. The majority of epidemiological studies use the living environment area classification method as an index of exposure, rather than assessing individual exposure. 

There is little research on the association between air pollution exposure with blood pressure levels in early pregnancy [5,8], but the few available studies suggest that pregnant women are a susceptible group for the studied blood pressure disorders. Reduction of air pollution in green areas has been suggested as a possible mechanism of the beneficial effects of green space [10]. Residence in proximity to green spaces may also have benefits for the health-related behaviour of urban residents—such as increased physical activity and social contacts [17,18], or psychological restoration and stress reduction [19,20,21].

Assessments of the relationships between exposure to natural outdoor environments and blood pressure are sparse, and the associations with the effects of different blood pressure categories remain uncertain [1,4,13,22]; besides, there are no such studies on pregnant women. There is no specific level of blood pressure at which cardiovascular complications start to occur. High-normal blood pressure is characterized as a level above the population level associated with increased risk for development of hypertension and cardiovascular events [23]. 

This study was conducted as part of the Positive Health Effects of the Natural Outdoor Environment in Typical Populations in Different Regions in Europe (PHENOTYPE) project funded by the European Commission Seventh Framework Programme (www.phenotype.eu [24]). The study formed part of the PHENOTYPE green space and health program [25].

To our knowledge, this is the first study to examine the association between green space and blood pressure groups in early pregnancy. In this paper, we hypothesised that the impact of green space on blood pressure amongst women depends on the proximity of the place of residence from the city park. Furthermore, we explored whether the associations of city park proximity differed in four blood pressure categories among the studied women, while controlling for confounding variables. 

## 2. Experimental Section

### 2.1. Study Population

This cross-sectional study was a part of a larger project on health effects of the natural outdoor environment (www.phenotype.eu [24]). The participants were pregnant women recruited to the European Commission’s FP6 HiWATE project between 2007 and 2009 in the city of Kaunas, Lithuania [26]. The women were recruited to the study in the early stages of pregnancy (97%—till 25 weeks). In total, 5,202 women were approached; 79% of them agreed to participate in the study. In this green space and blood pressure study, women with multiple pregnancies (150), those with inconsistent data on the estimation of exposure (mostly, students who moved out of the city during pregnancy, 405), or those for whom blood pressure was not measured during the first trimester of pregnancy (140) were excluded. The analyses here included 3,416 female residents of Kaunas, who were pregnant and were 20 to 45 years old at the time of the interview, and had at least one year of residence at their current address. The study was approved by the Lithuanian Bioethics Committee, and written informed consent was obtained from all participants.

### 2.2. Assessment of Blood Pressure and Confounders

The study participants’ blood pressure was measured by a well-trained physician. The blood pressure measurements were taken in a seated position, after five minutes of rest, using a mercury column sphygmomanometer placed on the right arm. The mean value of two blood pressure readings—systolic (SBP) and diastolic (DBP) blood pressure levels—over a two-minute interval was documented for each participant. The estimation of the blood pressure category was based on at least two blood pressure readings taken on two separate occasions. At baseline examination, women were classified into one of the four blood pressure categories based on the ESH-ESC 2007criteria: optimal (SBP < 120 mm Hg and DBP < 80 mm Hg), normal (SBP 120 to 129 mm Hg or DBP 80 to 84 mm Hg), high-normal blood pressure (SBP 130 to 139 mm Hg or DBP 85 to 89 mm Hg), and hypertension (SBD ≥ 140 or DBP ≥ 90 mm Hg) [27]. The normal blood pressure group, the high-normal blood pressure group, and hypertension group were compared with the optimal blood pressure (reference) group to identify risk factors for increased blood pressure. 

Pregnant women were asked to complete questionnaires provided to them at the clinic. The interview queried women regarding demographics, chronic diseases (cardiovascular, respiratory, renal, and diabetes), and duration of residence. Women reported their age at inclusion (less than 30 years or 30 years and older), education (primary, secondary, or university-level), social status (worker, student, unemployed—low; housekeeper, officer—medium; manager, company owner—high), marital status (married, not married), smoking (non-smoker or smokes at least one cigarette per day), alcohol consumption (0–1 drinks per week or 2 and more drinks per week), and other potential risk factors for hypertension. Body mass index (BMI) (<25, 25–30, >30) was calculated as weight (kg) divided by height squared (m^2^). The respondents’ perceived stress was measured by a question: “My daily activities are very trying and stressful”. The four response options were: “this describes me very well” (1), “fairly well” (2), “not very well” (3), or “not at all” (4), were scored with 1–4 points, and were used to define stress. Values 1 and 2 were considered to represent “stress”, and values 3 or 4—“no stress”.

### 2.3. Environmental Exposure Assessment

Spatial land cover data sets for the city of Kaunas were obtained from the municipality, and were processed using ArcGIS 10 software to produce the green space classification. Our definition of “green space” included all structured Kaunas municipality recreational places treated as City Parks larger than 1 ha; most of them had 65% of the area covered with trees. All the parks are open to public and offer similar recreation opportunities (e.g., walking, jogging, rollerblading, physical training, or resting on the bench) and locations (located among residential homes or establishments, and near public transport lines).

We used GIS to estimate the distance to the nearest park for 3,416 women at their current residential address. We determined three green space distance categories to the nearest park, using circular buffers with a radius of <300 m, 300–1,000 m, and >1,000 m around each woman’s place of residence. This choice was selected on the basis of European guidelines for access to recreation green spaces [28], and on the previous findings indicating that the impact of green environments on air pollution levels is often manifested in nearby neighbourhoods, and declines rapidly as the distance from green spaces increases. 

### 2.4. Statistical Analysis

We used chi-square tests to compare the values and frequencies of baseline characteristics by the blood pressure category of the women. Predictor variables whose univariate test showed a statistically significant association (*p* < 0.05) to the outcome were treated as possible risk factors for hypertension, and were included in the multinomial regression models. Multinomial regressions were used to assess the relationship between green space proximity as a categorical variable and as a continuous variable, and the blood pressure categories, adjusting for age, education, socioeconomic position, passive smoking, BMI, chronic disease, parity, and stress. The optimal blood pressure group was used as the reference group. The effects of green space on doctor-diagnosed blood pressure categories were estimated as crude and adjusted odds ratios (OR) with 95% confidence intervals (CI). All statistical analyses were performed using SPSS version 18.0 (SPSS Inc. Released 2009. PASW Statistics for Windows, Version 18.0., Chicago, IL, USA). 

## 3. Results and Discussion

### 3.1. Results

#### 3.1.1. Risk Factors for Hypertension

In this cross-sectional study, 3,416 women had complete blood pressure data and could be geocoded according to their residential address. The women who participated in the study were predominantly Lithuanian by their ethnic origin (97.4%), and were non-smokers (93.2%). The mean age was 28.4 years, and the women tended to be well-educated (54.6% of them—with a university degree). The prevalence of hypertension was 14.0%. Table 1 shows the distribution (%) of the women’s characteristics by blood pressure category. In general, some characteristics were unequally distributed (*p* < 0.05): education, marital status, socio-economic status, BMI, parity, passive smoking, chronic disease, and stress. We treated these variables as possible risk factors for hypertension. 

**Table 1:** Distribution (%) of women’s characteristics by blood pressure category.

Risk Factors for Hypertension	Blood Pressure Category ^a^			
	Optimal N ^c^ (%)	Normal N ^c^ (%)	High-normal N ^c^ (%)	Hypertension N ^c^ (%)
***Women’s age***				
<30 years	498 (23.8)	1,089 (52.0)	221 (10.6)	285 (13.6)
≥30 years	359 (27.1)	656 (49.6)	119 (9.0)	189 (14.3)
***Ethnic group***				
Lithuanian	834 (25.1)	1,693 (50.9)	333 (10.0)	466 (14.0)
Other	23 (25.5)	52 (57.8)	7 (7.8)	8 (8.9)
***Education ^b^***				
Primary school	44 (26.2)	98 (58.3)	10 (6.0)	16 (9.5)
Secondary school	308 (22.2)	737 (53.1)	119 (8.6)	223 (16.1)
University degree	505 (27.2)	910 (48.9)	211(11.3)	235 (12.6)
***Marital status***				
Married	716 (25.5)	1,435 (51.0)	279 (9.9)	384 (13.6)
Not married	141 (23.4)	310 (51.5)	61 (10.1)	90 (15.0)
***Socio-economic status ^b^***				
Low	224 (22.2)	544 (53.9)	85 (8.4)	157 (15.5)
Medium	488 (26.8)	906 (49.7)	195 (10.6)	235 (12.9)
High	133 (26.2)	255 (50.3)	52 (10.3)	67 (13.2)
***Body mass index (kg/m^2^) ^b^***				
<25 Underweight and normal	639 (31.8)	1,017 (50.7)	172 (8.6)	179 (8.9)
25–30 Overweight	175 (18.0)	532 (54.8)	116 (12.0)	147 (15.2)
>30 Obesity	43 (9.8)	196 (44.7)	52 (11.8)	148 (33.7)
***Parity ^b^***				
No child	344 (22.5)	790 (51.8)	170 (11.1)	223 (14.6)
≥1 child	513 (27.1)	955 (50.6)	170 (9.0)	251 (13.3)
***Women’s active smoking***				
Non-smoker	800 (25.1)	1,628 (51.2)	306 (9.6)	449 (14.1)
Smoker	57 (24.5)	117 (50.2)	34 (14.6)	25 (10.7)
***Passive smoking ^b^***				
No	500 (27.9)	886 (49.5)	169 (9.4)	236 (13.2)
Yes	350 (22.0)	836 (52.6)	170 (10.7)	234 (14.7)
***Chronic disease ^b^***				
No	613 (23.8)	1,351 (52.3)	267 (10.3)	351 (13.6)
Yes	244 (29.3)	394 (47.2)	73 (8.8)	123 (14.7)
***Alcohol consumption***				
No	798 (24.9)	1,636 (50.9)	325 (10.1)	453 (14.1)
Yes	59 (28.9)	109 (53.4)	15 (7.4)	21 (10.3)
***Stress ^b^***				
No	611 (24.6)	1,293 (52.1)	229 (9.2)	351 (14.1)
Yes	246 (26.4)	452 (48.5)	111 (11.9)	123 (13.2)

Notes: ^**a**^ Optimal blood pressure: SBP < 120 mm Hg and DBP < 80 mm Hg; normal blood pressure: SBP 120–129 and DBP 80–84 mm Hg; high-normal blood pressure: SBP 130–139 and DBP 85–89 mm Hg; and hypertension: SBD ≥ 140 or DBP ≥ 90 mm Hg. **^b^** *p* < 0.05. **^c^** Values are the number of subjects (percentage) for categorical variables.

#### 3.1.2. Distribution of the Studied Population by Exposure to Green Space

Table 2 shows distribution (%) of the residents’ characteristics by categories of proximity to city parks (<300 m, 300–1,000 m, and >1,000 m). 

**Table 2:** Distribution (%) of women’s characteristics according to green space distance categories.

Characteristics	Green Space Distance Category ^a^		
	<300 m N ^c^ (%)	300–1,000 m N ^c^ (%)	>1,000 m N ^c^ (%)
***Women’s age***			
<30 years	535 (62.5)	1,254 (61.0)	304 (59.6)
≥30 years	322 (37.5)	803 (39.0)	198 (39.4)
***Marital status***			
Married	718 (83.8)	1,697 (82.5)	399 (79.5)
Not married	139 (16.2)	360 (17.5)	103 (20.5)
***Education***			
Primary school	39 (4.6)	101 (4.9)	28 (5.6)
Secondary school	380 (44.3)	804 (39.1)	203 (40.4)
University degree	438 (51.1)	1,152 (56.0)	271 (54.0)
***Active smoking***			
Non-smoker	795 (92.8)	1,918 (93.2)	470 (93.6)
Smoker	62 (7.2)	139 (6.8)	32 (6.4)
***Passive smoking***			
Non-smoker	436 (51.7)	1,093 (53.6)	262 (52.7)
Smoker	407 (48.3)	948 (46.4)	235 (47.3)
***Alcohol consumption***			
No	810 (94.5)	1,936 (94.1)	466 (92.8)
Yes	47 (5.5)	121 (5.9)	36 (7.2)
***Blood pressure***			
<120/80 mmHg	225 (26.3)	527 (25.6)	105 (20.9)
120-129/80-84 mm Hg	434 (50.6)	1,046 (50.9)	265 (52.8)
130-139/85-89 mm Hg	76 (8.9)	199 (9.7)	65 (12.9)
≥140 or ≥90 mm Hg	122 (14.2)	285 (13.9)	67 (13.3)
***Ethnic group***			
Lithuanian	832 (97.1)	1,997 (97.1)	497 (99.0)
Other	25 (2.9)	60 (2.9)	5 (1.0)
***Parity ^b^***			
No child	400 (47.8)	917 (44.6)	200 (39.8)
≥1 child	447 (52.2)	1,140 (55.4)	302 (60.2)
***Chronic disease***			
No	648 (75.6)	1546 (75.2)	388 (77.3)
Yes	209 (24.4)	511 (24.8)	114 (22.7)
***Socio-economic status***			
Low	263 (31.6)	597 (29.6)	150 (30.4)
Medium	449 (54.0)	1,120 (55.6)	255 (51.6)
High	120 (14.4)	298 (14.8)	89 (18.0)
***Body mass index (kg/m^2^)***			
<25 Underweight and normal	525 (61.3)	1,199 (58.3)	283 (56.4)
25–30 Overweight	225 (26.3)	592 (28.8)	153 (30.5)
>30 Obesity	107 (12.5)	266 (12.9)	66 (13.1)
***Maternal stress***			
No	644 (75.1)	1,477 (71.8)	363 (72.3)
Yes	213 (24.9)	580 (28.2)	139 (27.7)

Notes: ^**a**^ Distance to the nearest city park. **^b^** *p* < 0.05. **^c^** Values are the number of subjects (percentage) for categorical variables.

Study participants were evenly distributed by distance to green spaces according to age, marital status, maternal stress, education, smoking, ethnicity, and other variables, but not for parity. The proportion of women with optimal blood pressure tended to be higher in the area nearest to the park (26.3%) than in the farthest area (20.9%), while the proportion of women with high-normal blood pressure tended to be higher in the area farthest to the park than in the nearest to the park area (12.9% and 8.9%, respectively). Overweight and maternal stress tended to increase with increasing distance between the place of residence and the nearest city park.

#### 3.1.3. Relationship between Green Space Exposure and Blood Pressure

Table 3 shows the associations between green space proximity and blood pressure categories among the subjects of the study at different levels of analysis. 

**Table 3:** Crude and adjusted odds ratios (OR) and 95% confidence intervals (CI) for increased blood pressure according the proximity to city parks.

Blood Pressure Categories	Green Space Distance	Cases N (%)	Crude		Adjusted	
			OR	95% CI	OR ^a^	95% CI
Normal 120–129/ 80–84 mmHg	<300 m	434 (24.9)	1		1	
	300–1,000 m	1046 (59.9)	1.03	0.85–1.25	1.06	0.87–1.29
	>1,000 m	265 (15.2)	1.31	0.99–1.73	1.37	1.03–1.82
	Continuous **^b^**		1.08	1.01–1.15	1.09	1.02–1.17
High-normal 130–139/ 85–89 mmHg	<300 m	76 (22.4)	1		1	
	300–1,000 m	199 (58.5)	1.12	0.82–1.52	1.10	0.80–1.51
	>1,000 m	65 (19.1)	1.83	1.22–2.75	1.74	1.14–2.66
	Continuous **^b^**		1.14	1.04–1.27	1.14	1.02–1.26
Hypertension ≥140 or ≥90 mmHg	<300 m	122 (25.8)	1		1	
	300–1,000 m	285 (60.1)	1.00	0.77–1.30	1.01	0.76–1.35
	>1,000 m	67 (14.1)	1.18	0.81–1.72	1.18	0.79–1.77
	Continuous **^b^**		1.02	0.93–1.12	1.02	0.93–1.13

Notes: OR (crude odds ratios) are based on multinomial regression models, and reflect the risk for an increase in blood pressure category depending on the distance to city parks, compared to the reference group (optimal blood pressure <120/80 mm Hg). **^a^** OR adjusted for age, education, socioeconomic position, passive smoking, BMI, chronic disease, parity, and stress. **^b^** OR increase per every 300 m increase in the distance to city parks.

In univariate analysis (crude OR), distance to green spaces was positively related to the normal blood pressure category, compared to the reference category (optimal blood pressure <120/80 mm Hg). With increasing distance to city parks, the increase in the odds ratios for high-normal blood pressure was statistically significant; the crude ORs were 1.12, 95% CI 0.82–1.52 and 1.83, 95% CI 1.22–2.75, respectively, for moderate and the farthest distance *vs.* the nearest distance to city parks. We did not find any statistically significant association between the proximity of city parks and the hypertension group: the crude ORs were 1.00, 95% CI 0.77–1.33 and 1.18, 95% CI 0.81–1.72, respectively, for moderate and the farthest distance *vs.* the nearest distance to city parks.

Adjusting for age, education, socioeconomic position, passive smoking, BMI, chronic disease, parity, and stress did not substantially change the odds ratios, showing statistically significant results for the normal and high-normal blood pressure categories (1.37, 95% CI 1.03–1.82 and 1.74, 95% CI 1.14–2.66 for the farthest *vs.* the nearest distance, respectively). The association between the proximity of city parks and the hypertension group was not statistically significant: the adjusted ORs were 1.18, 95% CI 0.79–1.77 for the farthest *vs.* the nearest distance, accordingly. The analysis of park proximity as a continuous variable also showed statistically significant results for the normal and high-normal blood pressure groups. The adjusted ORs for high-normal blood pressure group increased by 1.14, 95% CI 1.02–1.26 for every 300 m increase in the distance between the place of residence and city parks.

### 3.2. Discussion

In this cross-sectional study, univariate and multivariate statistical analysis models revealed that the proximity of the place of residence to green spaces was associated with lower blood pressure among pregnant women. We observed statistically significant positive relationships of the prevalence of optimal-normal blood pressure and high-normal blood pressure with the distance of the place of residence from city parks. Using the optimal blood pressure as the reference group, the adjusted odds ratio (OR) for normal blood pressure and for high-normal blood pressure were statistically significant higher after the inclusion of the selected covariates in the categorical multinomial regression models. The analysis of continuous variables also showed an increase in the risk for normal blood pressure group by 9% and for high-normal blood pressure group ‒ by 14% for every 300 m increase in the distance from green spaces. However, the association between proximity to city parks and the hypertension group was not statistically significant.

To address the question regarding if and how much green space as an independent factor contributes to blood pressure differences during first trimester of pregnancy, we used data from the well-characterised study of pregnant women of the city of Kaunas. Women during the first trimester of pregnancy are a suitable group for the study of hypertensive disorders, since even though changes in pregnancy lead to an increased stress on the cardiovascular system, such effects mainly occur from the second trimester of pregnancy on [29]. Therefore, blood pressure during the first trimester of pregnancy is mainly associated with external factors. 

In this study, the impact of residential proximity to city parks on the doctor-diagnosed increased blood pressure were estimated by multinomial regression models with adjustments for age, education, body mass index, passive smoking, chronic diseases, alcohol consumption, and parity. We observed an impact on blood pressure even when the distance to a city park was included simultaneously in the model together with other individual covariates. The estimated impact showed an exposure-response pattern. 

Our results may show random variation; however, distance estimation on the individual level, control for the subjects’ migration effect, a strict protocol for measuring blood pressure, and control for possible confounding suggest that green space might contribute to blood pressure differences as an independent factor. This study is the first to investigate the association between residential proximity to city parks and the risk of increased blood pressure among pregnant women.

Previous epidemiological research has found direct relationship between the blood level and the occurrence of cardiovascular events [30]. A number of potential mechanisms have been suggested to explain the beneficial effects of living near green space (including increased recreational physical activity [18,31] and social contacts [17]) on the cardiovascular system and physiological stress [32,33]. Furthermore, the results indicated that the shorter the distance was from the respondents’ homes to the nearest green space, the less stress they experienced [34,35].

Other publicised data showed that the availability of recreational spaces—such as parks—is associated with better perceived overall health [36], higher levels of physical activity, and lower levels of overweight and obesity [37,38]. The study conducted in the city of Bristol used objectively measured access to the city park, and showed that the reported frequency of green space use declined with an increasing distance [21]. This study also found that respondents living closest to the Formal Park were more likely to achieve the recommendations for physical activity. 

Our results are in accordance with other studies in which no statistically significant association was observed between access to parks and the prevalence of obesity or overweight [39,40], or stress [34], which are the primary risk factors of hypertension. Unfortunately, we did not have any information on the subjects’ physical activity and that might have an impact on our results. Adjustment for stress did not change our risk estimates materially, suggesting that other mechanisms may be responsible for the observed effects.

To our knowledge, only few published studies investigated the association between green environment and blood pressure using objective environmental characteristics [12]. The authors used satellite remote sensing data to characterise the land cover/land use environment for the studied areas. The authors found that in urban areas, the incidence of systolic and diastolic hypertension was higher than in it was suburban or rural areas. However, adjustment for cardiovascular risk factors attenuated this association.

Study findings from the Netherlands showed that neighbourhood-level environmental stressors were associated with blood pressure in ethnic minority groups, but the association was less evident in the Dutch group [40]. The authors found that “high quality” of green spaces and social participation were associated with a lower systolic blood pressure, but that study did not include any control for internal migration effects.

Strength of the present population-based study is that we individually estimated green space exposure to the participants with the same ethnic culture and the health care system. Additional strengths include the possibility to control for potential confounding factors and the covariates of hypertension. Misclassifications of increased blood pressure cases were not likely, as physician-diagnosed blood pressure is generally considered to be reliable. Therefore, this study provides valid data for assessing the associations between proximity to city parks and blood pressure. We assigned increased blood pressure cases independently of environmental exposure; however, non-differential classification errors may have tended to underestimate the effects of the distance to green spaces [41], and the possibility that an increased blood pressure may result from variables not controlled for in this study cannot be excluded either. There are a number of limitations associated with our study. This was a cross-sectional study, and as such could not identify a causal relationship. We do not have data on actual green space use, and did not take into account other types of exposure associated with the studied women’s activity (e.g., indoor, occupational, or commuting). In this study, we did not examine individual women’s susceptibility to environmental exposure. Our previously published data indicate that the magnitude of the response to environmental exposures depends on individual susceptibility and gene polymorphisms [42]. Genetic variation at the angiotensinogen locus and G protein polymorphisms may affect individual susceptibility to developing essential hypertension through a variety of physiological mechanisms [41,43] and interactions among genetic and environmental factors. 

Previously published data showed [44] that compared with optimal blood pressure, high-normal blood pressure is a risk factor for stroke and myocardial infarction in the general urban population. The study suggests that high-normal blood pressure is associated with a shortened life expectancy. In a most cases the hypertension disease develops gradually and blood pressure levels of “normal” blood pressure category and “high-normal” blood pressure category should be treated as early stages of hypertension that might be controlled by behavioral population-based interventions. Therefore, to establish public policy for hypertension and cardiovascular disease prevention, further environmental epidemiological studies should consider the contribution of both the environmental factors and the internal factors on the study subjects [45], and should comprise early stages of hypertension.

## 4. Conclusions

In conclusion, the findings of the present study extend the knowledge base with objective measures of the effect of green spaces on blood pressure elevation, and have an important public health application in supporting green urban planning policy, prioritising health, and preventing hypertension. Our results suggest that proximity to green spaces may positively affect women’s blood pressure. This association seemed to be independent of other known risk factors of hypertension. Further studies are required to explore the effects of the magnitude of green space exposure-response modifying factors (such as genetic susceptibility) on the course of hypertension, physical activity, use of recreational greenery and to explore the underlying mechanisms. 
